# Family Supportive Leadership and Counterproductive Work Behavior: The Roles of Work-Family Conflict, Moral Disengagement and Personal Life Attribution

**DOI:** 10.3389/fpsyg.2022.906877

**Published:** 2022-05-27

**Authors:** Shan Jin, Xiji Zhu, Xiaoxia Fu, Jian Wang

**Affiliations:** ^1^Business School, Central University of Finance and Economics, Beijing, China; ^2^School of Information, Central University of Finance and Economics, Beijing, China

**Keywords:** counterproductive work behavior, family supportive leadership, moral disengagement, personal life attribution, work-family conflict

## Abstract

Counterproductive work behavior (CWB) is one of the most common behavioral decisions of employees in the workplace that negatively impacts the sustainable development of enterprises. Previous studies have shown that individuals make CWB decisions for different reasons. Some individuals engage in CWB due to cognitive factors (i.e., perceived organizational justice and psychological contract breakdown), whereas others engage in CWB in response to leadership behaviors (i.e., abusive management). The conservation of resources (COR) theory holds that individuals have the tendency to preserve, protect and acquire resources. When experiencing the loss of resources, individuals will show irrational and aggressive behaviors in order to regain resources. When obtaining resources, individuals’ tension and pressure will be relieved. To maintain or continue obtaining resources, individuals will show more positive work attitudes and behaviors. Therefore, using the COR theory as the main theoretical framework, this study explores a serial mediation model between family supportive leadership and CWB through work-family conflict and moral disengagement, moderated by personal life attribution. A three-wave survey of 251 medical workers from three hospitals found that family supportive leadership can reduce employees’ perceived work-family conflict, which leads to less moral disengagement, resulting in lower CWB. Personal life attribution strengthens the negative indirect effect of family supportive leadership on CWB by reinforcing the negative association between family supportive leadership and work-family conflict. This study uses the COR theory to explore the mechanism and boundary conditions of family supportive leadership and CWB from the perspective of negative work-family relationship, which enrichis the research content of existing theories. Moreover, this study has important guiding significance for managers to take effective measures to reduce CWB.

## Introduction

Employees make some decisions, such as unethical ones, that seriously harm the organization’s long-term interests. In particular, CWB refers to the behavior that deliberately violates important organizational norms, policies, or systems in the workplace and affects the well-being of the organization or its members, including interpersonal and organizational deviance (Murad et al., 2021; Pletzer, 2021). Estimates show that about 35%?55% of employees decide to perform negative workplace behaviors, including abuse of office equipment and sick days, company property theft, sabotage, interpersonal rudeness, and absenteeism (Umphress et al., 2010). These negative behaviors produce organizational losses estimated to range from $6 to $200 billion annually (Christian and Ellis, 2011).

Employees’ CWB may disturb the effective operation of the enterprise, cause property losses and tension with colleagues, or undermine team cohesion and work efficiency (Shelly et al., 2018; De Clercq et al., 2019). However, the question “Why do employees often make such unethical decisions?” is yet to be answered. Some scholars have shown that moral disengagement is considered one of the strongest predictors of CWB (Detert et al., 2008; Barsky, 2011; Moore et al., 2012). When resources are threatened or lost, individuals may reduce their moral self-control, resulting in the psychological mechanism of moral disengagement, including excuses for their unethical decision-making behavior. As moral awareness and moral initiative are constrained, individuals will engage in unethical behavior to obtain resources (Kish-Gephart et al., 2010). Existing studies have explored the antecedents of moral disengagement and CWB, specifically including personality (Li et al., 2020; Mackey et al., 2021), stressors (Fida et al., 2014), leadership (Brender-Ilan and Sheaffer, 2019; Moore et al., 2019), collective perceptions of the work environment (Nichelle et al., 2021), perceptions of organizational politics (Valle et al., 2019), and job insecurity (Huang et al., 2017). These findings suggest that when employees’ moral disengagement increases, the scope of allowable deviant behaviors will also increase.

In addition, regarding the influencing factors of moral disengagement and CWB, most empirical studies are conducted to investigate single factors such as employee personality characteristics and leadership style (Li et al., 2020; Murad et al., 2021). However, it is yet to be clarified whether the interference between the work and non-work domain will also cause employees’ moral disengagement and CWB, such as work-family conflict (Pletzer et al., 2019; Selvarajan et al., 2019). In China’s unique cultural background, the relationship between work and family is inseparable, and the two fields often overlap. Especially since the beginning of the 21st century, there have been increasing dual-worker and single-parent families, as well as employees with multiple family care responsibilities, thus aggravating work-family conflict (Asghar et al., 2018). Employees may try to earn money to support their families, sacrifice work opportunities, and behave in ways that do not align with organizational norms. In particular, when work interferes with family, employees’ moral psychology and behavior change. Since work drains employees’ time and energy to bond with family, they may rebel and demonstrate immoral behaviors in the organization. Work-family conflict could also cause depletion of employees’ resources. To replenish resources or relieve the role pressure, employees experience moral disengagement. This will impede their self-regulation mechanism, enabling unethical activities, such as CWB (Lee et al., 2016). Thus, work-family conflict may also be an important antecedent of moral disengagement and CWB. However, existing studies have ignored this critical factor.

Moreover, existing studies have found that work-family conflict negatively impacts employees, families, and organizations, improving turnover intention and reducing job satisfaction (French et al., 2018). To resolve these issues, Hammer et al. (2009) proposed a new leadership style of family supported leadership. Empirical studies have proved that family supportive leadership can effectively reduce the adverse effects of work-family conflict, job withdrawal behavior, and turnover intention (Kailasapathy and Jayakody, 2018; Pan, 2018; Han and McLean, 2020; Nguyen et al., 2020). Family supportive leadership can positively influence employees’ psychology and reduce employees’ negative behaviors. In addition, employees’ varying degrees of personal life attribution to leadership behavior may also affect the effectiveness of family supportive leadership. Therefore, this study considers family supportive leadership a vital method to solve employees’ work-family conflict, moral disengagement and CWB, while discussing the influence of personal life attribution as a crucial boundary condition.

Accordingly, this study uses the COR theory was proposed by Hobfoll (1989) to include family supportive leadership and work-family conflict into the study of moral disengagement and CWB, and explore the internal serial mediating effect of work-family conflict and moral disengagement on family supportive leadership and CWB as well as the influence of personal life attribution as a boundary condition on the effect of family supportive leadership. This expands the research scope of existing literature. Conservation of resources believes that resource loss will be accompanied by tension and stress response, and individuals will try to take actions to avoid resource loss. Moreover, in the context of resource loss, individuals’ defense mechanisms will be triggered and some aggressive and irrational behaviors will be displayed. COR makes assumptions about subsequent cognitive and behavioral mechanisms of resource gain and loss. The loss of resources caused by work-family conflict and the resources generated by family supportive leadership will affect employees’ psychological and behavioral responses. The loss and increase of resources are the theoretical basis for understanding the internal mechanism of employee psychological and behavioral decision changes. Therefore, COR should be used to explain the internal mechanism of family supportive leadership and CWB.

This paper has three main purposes. First, we expand the scope of CWB research by exploring the antecedents of CWB from the perspective of work and family relationships. Employees have to manage the relationship between work and family continuously. Moreover, the conflict between work and family drains employees’ physical and psychological resources (De Clercq et al., 2019). The absence of family life, family members’ complaints, and the guilt of not being able to undertake family responsibilities further aggravates their psychological pressure. This leads to negative psychological states and immoral behaviors such as job burnout and workplace aggression among employees (French et al., 2018).

Furthermore, such negative psychological states and immoral behaviors may cause employees to verbally or physically assault others, abuse organizational resources, conduct non-essential activities during work hours, and engage in workplace deviant behaviors (Walsh et al., 2018; Zhang Y. et al., 2019). According to the COR theory, individuals experiencing resource loss have greater difficulty performing effective resource investment activities (Newman et al., 2019). Thus, to compensate for the shortage of existing resources and cope with the work-life conflict, individuals will engage in deviant and immoral behaviors while defending such behaviors (He et al., 2019).

Second, this study examines whether family supportive leadership will affect employees’ moral psychology and behavioral decision-making in the workplace by reducing employees’ work-family conflict. It is crucial to identify the antecedents that lead to moral disengagement and CWB as well as determine ways to solve the problems of work and non-work life, reduce the negative impact on employees’ follow-up behavior, and enable employees to devote better themselves to work (Newman et al., 2019). Based on the COR theory, leaders’ support resources are essential sources of employees’ resources. Family supportive leadership provides employees with corresponding resources to deal with work and family problems, such as communicating with employees and providing corresponding suggestions and flexible working hours to reduce work interference on family affairs (Kelly et al., 2020). This form of support may cause employees to feel grateful for their leaders’ support, enabling them to engage in activities that are beneficial to the organization or try to avoid causing damage to the organization’s reputation (Walsh et al., 2018).

Third, this study explores personal life attribution as the boundary condition for the impact of family supportive leadership on employees’ work attitudes and behavior. Since family supportive leadership behavior is a kind of informal support behavior outside the role of leadership, employees may make attributions to their leaders’ actions. According to the COR theory, when employees have a higher degree of attribution to the personal life of the family supporting leader, employees will perceive their leader’s support resources as desirable and accept their support and resources; further, the leadership behavior will have a more substantial effect on the work-family conflict of employees. To maintain and obtain more leadership support resources, employees will try to engage in positive behaviors (Hobfoll et al., 2018). With certain resources, employees may not have negative psychological states and workplace behaviors. Suppose employees attribute the leader’s family support behavior to helping them balance the demands of work and non-work life. In that case, employees will believe that the leader genuinely wants to help employees cope with work and family problems and provide resources to relieve the tension and pressure caused by work-family conflict. When employees use leaders’ support resources, they will not worry about the negative impact on their career development, and the effect of leaders’ support resources on work attitude and behavior will be more significant. Consequently, employees will unlikely perform unethical behaviors in the workplace to continue receiving supportive resources from their leader.

**FIGURE 1:**
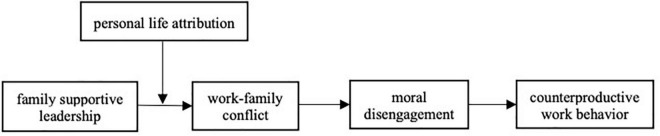
The comprehensive model.

Across medical workers from hospitals, we use three-wave data to evaluate the influence of family supportive leadership on employees’ CWB. The changes in employee CWB allow us to begin to assess the broader impact that family supportive leadership may have and whether low levels of work-family conflict and low moral disengagement associated with family supportive leadership behavior lead employees to engage in less CWB. Our study contributes to existing research on the broader effects of family supportive leadership on CWB through serial mediating employees’ work-family conflict and moral disengagement. We also expand the existing literature by introducing personal life attribution as a boundary condition. Our results suggest that work-family conflict and moral disengagement play important roles in the relationship between family supportive leadership and CWB and that personal life attribution can influence the effect of family supportive leadership (the comprehensive model is shown in Figure 1).

## Theoretical Background

### Conservation of Resources Theory

This study uses COR theory as the theoretical basis to explore how family supportive leadership can reduce employees’ CWB (Hobfoll et al., 2018). Conservation of resources puts forward that individual resources are limited. When facing the threat of potential resource loss or actual resource loss, the defense mechanism of individual self-protection will be triggered and show some aggressive and irrational behaviors. Work-family conflict will cause the loss of employees’ psychological and physiological resources. At this time, to avoid further loss of resources or regain lost resources, employees will show moral disengagement psychology and immoral workplace behavior.

The COR holds that the acquisition or loss of resources at work will affect the psychological state and behavior of employees (Hobfoll et al., 2018). Leader is an important source of workplace resources for employees, which can indicate whether employees have received resource help and support when facing the multiple role needs of work and family. As an informal leadership support type outside the role, family supportive leadership can give corresponding resource support according to the specific needs of employees. This will alleviate the conflict between employees’ work needs and family needs. After the work-family conflict is alleviated, the loss of resources will be improved. To obtain more leadership support resources, employees will reduce their moral shirking psychology and deviant behavior in the workplace to improve work efficiency and team cohesion.

## Hypotheses

### Moral Disengagement and Counterproductive Work Behavior

Moral disengagement refers to the process in which employees do not follow the moral standards in work or life and deliberately conduct immoral behaviors. When employees engage in moral disengagement, they explain their unethical behaviors without feeling remorse (Newman et al., 2019). Bandura (1999) put forward a set of interrelated moral mechanisms, including eight aspects, such as moral justifications, euphemistic labeling, advantageous comparison, displacement of responsibility, diffusion of responsibility, disregard or distortion of consequences, dehumanization, and attribution of blame (Bandura, 1999; Keem et al., 2018). According to the COR theory, employees engage in moral evasion due to the loss or shortage of resources. To obtain more resources or compensate for the shortage of existing resources, employees defend and recourse to sophistry for their immoral behavior. Since they are not psychologically troubled by misbehavior, they attempt to justify their misbehavior by making it seem morally acceptable, thus deviating from the norm at work and engaging in CWB (Pletzer et al., 2019; Chen et al., 2020).

Moreover, the cognitive mechanism of moral disengagement helps cognitively reconstruct unethical behavior, making it seem less harmful, thus increasing CWB (Spector and Fox, 2010; Zheng et al., 2019). This effect is more apparent when employees have a high turnover intention (Zhang et al., 2020). Employees tend to commit minor mistakes that can easily be proven reasonable, and moral disengagement may make a person who commits small mistakes defend future deviant behaviors. Employees promote moral disengagement in the workplace through progressive induction mechanisms, in which deviant behavior becomes routine over time and is seen as acceptable without additional consideration. Moral disengagement can explain why people tend to engage in a series of progressively increasing transgressions and are therefore easier to rationalize than a sudden increase in transgressions (Welsh et al., 2015). If employees have the psychological tendency of moral disengagement, their internal moral self-regulation may decline, causing them not to feel guilty and self-blame when they engage in deviant workplace behaviors (Knoll et al., 2016). Thus, we propose the first hypotheses:

*Hypothesis 1. Moral disengagement will positively impact counterproductive work behavior.*

### Work-Family Conflict and Moral Disengagement

Work-family conflict refers to an interrole conflict in which employees cannot meet work and family demands simultaneously due to resource limitations (Asghar et al., 2018). This conflict is caused by the fact that neither of the two roles has enough resources to meet requisite needs. Employees lack time or energy to perform their family duties due to heavy workload or performance pressure, leading to work-family conflict. If employees’ work-family conflict is not timely alleviated and handled, it will negatively impact the organization and individual employees by affecting employees’ work performance and sleep quality, increasing the possibility of dismission, and worsening family relationships (Perry-Jenkins and Gerstel, 2020). The primary source of work-family conflict is mainly the lack or uneven distribution of time and energy. Resource limitations resulting from work-family conflict may increase psychological pressure, causing adverse consequences such as resignation tendency, burnout, complaints, work-related injuries (Netemeyer et al., 1996; Halbesleben, 2010), and work-family conflict. To save the limited resources or obtain more resources, employees experience moral disengagement, justify and find reasons for their immoral behavior, and make their behavior seem reasonable (Pluut et al., 2018). Thus, we propose the second hypothesis:

*Hypothesis 2. Work-family conflict will positively impact moral disengagement.*

### Family Supportive Leadership and Work-Family Conflict

Family supportive leadership refers to the leader’s efforts to make employees realize a virtuous work and family life cycle. They demonstrate understanding and support for the difficulties encountered by employees in the work process and provide help for the problems encountered by employees in their non-work life. Family supportive leadership is an informal type of leadership support, which is greatly influenced by individual factors of leaders (Crain and Stevens, 2018). It aims to balance the relationship between work and family so that employees can fulfill work requirements and consider their family’s needs. General leadership support behavior is not specifically for the employee’s family or life needs. Employees should independently deal with the relationship between work and family since managing family problems is beyond the supervisor’s responsibility (Rofcanin et al., 2017; Ahmad et al., 2020). Therefore, family supportive leadership is more in line with the organization’s long-term development, which is more prevalent among employees (Hammer et al., 2013; Straub et al., 2019).

Family supportive leadership places a high value on work and family problems reported by employees and in the process of working through a variety of ways to actively help employees deal with the work-family relationship, such as creative work-family management (Rofcanin et al., 2020). These support resources can help employees relieve mental pressure and meet their social and emotional needs to ensure that they have more time and energy to deal with work and family issues and better control of work-family balance (Rofcanin et al., 2018). Existing studies have proved that employees’ flexibility and ability to manage working time negatively correlate with work-family conflict (Pan et al., 2020). According to the COR theory, individuals must constantly protect existing resources from loss through resource investment, recover from resource loss faster, and obtain new resources. Family supportive leadership provides support and help resources to employees’ work and family life and communicates with them actively (Peng et al., 2020). To a certain extent, this makes up for the shortage of existing resources of employees and allows employees to have adequate physical and psychological resources to deal with problems in their work and non-work life (Marescaux et al., 2020). Providing employees with certain work or family support resources will enable and motivate them to generate more personal resources while preventing them from depleting resources due to work demands, thus helping reduce work-family conflict (Butts et al., 2013). Hence, we propose the third hypothesis:

*Hypothesis 3. Family supportive leadership will negatively impact work-family conflict.*

### The Serial Mediating Role of Work-Family Conflict and Moral Disengagement

Finally, we posit that work-family conflict and moral disengagement serially mediate the relationship between family supportive leadership and CWB. Existing studies show that work-family conflict negatively influences organizational development and employees’ subsequent work attitudes (Perry-Jenkins and Gerstel, 2020). To ease the conflict between job requirements and family responsibility, family supportive leadership encourages employees to use formal organizational welfare policy, provides personal care and support, and enables them to apply their own resources to cope with the work-family pressure (Marescaux et al., 2020). Moreover, family supportive leadership is good at listening and understanding employees’ family needs and can formulate effective work-family management strategies according to specific situations and provide flexible working hours, relevant information, and suggestions (Pan et al., 2020). This increases employees’ resources and reduces the loss of resources caused by work and family problems; through the influence on employees’ cognition, employees understand and realize that moral disengagement is wrong, thus reducing CWB (Bakker and Demerouti, 2017; Kelly et al., 2020).

When employees encounter an emergency, family supportive leadership can help employees coordinate relevant resources to complete work tasks smoothly. These support activities provided by family supportive leadership will affect employee behavior and practices (Rofcanin et al., 2018; Straub et al., 2019). Family supportive leadership helps develop and retain personal, emotional, and psychological resources, improving employees’ participation in relevant tasks and activities without worrying about other negative effects (Zhou et al., 2018; Suseno et al., 2021). For example, employees can openly and freely communicate with their superiors about family related issues (Hammer et al., 2016). Therefore, this study believes that under the influence and impetus of family supportive leadership, the individual resources of employees are supplemented, which reduces work-family conflict. To maintain the existing resources or obtain more resources, employees tend to develop or improve their internal moral standards and codes of conduct, and individual behavior becomes consistent with their moral principles. Once moral disengagement for negative behavior occurs, employees control their thoughts and behaviors through self-regulation, thus reducing moral disengagement (Newman et al., 2019). Thus, we propose the fourth hypothesis:

*Hypothesis 4. Work-family conflict and moral disengagement will serially mediate the relationship between family supportive leadership and counterproductive work behavior.*

### The Moderating Role of Personal Life Attribution

Personal life attribution refers to employees attributing leaders’ family support behaviors to perceptions that help employees deal with or adapt to non-work activities (Leslie et al., 2012; Shi et al., 2019). Employees have different attributions to the organizational service and self-service of family support leadership, and the effect of family support leadership behavior on employees’ work attitude and behavior may also vary. According to the COR theory, personal life attribution will affect the strength of the family supportive leadership effect (Lee and Carpenter, 2018; Walsh et al., 2018). At work, employees’ views on organization or leadership support are very important, which will affect employees’ acceptance of leadership support behavior and their views on leadership behavior. If employees attribute the leader’s family support behavior to genuine care and help for their personal life, this idea will play a synergistic role with the leader’s support behavior, and the leader’s support resources will be transformed into the employee’s available resources, thus reducing the degree of work-family conflict. We believe that the positive influence of family supportive leadership can be extended to employees with personal life attribution because they feel that the help and support provided by the supervisor consider their actual needs, When employees think that the leader’s family support behavior is sincere for their work and family interests and help them solve work family conflicts, employees will feel that they have received the care and help of the leader and will take the initiative to pull in the psychological distance from the leader. Causing them to be more willing to communicate with leaders and accept their support resources, which will not feel negative impact on their career (Halbesleben, 2010). After the work-family conflict is alleviated, to save existing resources or prevent the loss of resources, employees will be more proactive and loyal in the follow-up work, and will not produce some bad ideas and behaviors for the organization and leaders. Studies have shown that resources acquired in a field are more likely to have an enhancement effect if they are consistent with individual perception (Russo et al., 2018). Therefore, this study holds that family supportive leadership has a more decisive influence on employees’ CWB through work-family conflict and moral disengagement in the context of employees’ personal life attribution. Therefore, we propose the fifth hypothesis:

*Hypothesis 5. Personal life attribution will positively moderates the relationship between family supportive leadership and work-family conflict; that is, the higher the degree of personal life attribution, the stronger effect of family supportive leadership on employees’ work-family conflict.*

## Materials and Methods

### Participants and Procedure

This study collected data from medical workers in three hospitals in Beijing and Zhengzhou through electronic questionnaires. First, to obtain the support and cooperation of medical workers, we contacted the hospital’s functional departments and explained the purpose and significance of this study to the head of departments. The questionnaires were given to the departments, which they then sent to subordinates through email to ensure data confidentiality. To achieve the matching of samples, each employee was required to fill in the last four digits of their mother’s telephone number in the questionnaire code. This study used an anonymous method to complete the questionnaire in three stages, and the interval of each stage was one month. In the first stage, 300 questionnaires were distributed, employees were asked to complete basic personal information, evaluate family supportive leadership behavior and work-family conflict, and 292 valid questionnaires were collected. In the second stage, 300 questionnaires were distributed, employees were asked to complete moral disengagement and personal life attribution, and 283 questionnaires were collected. In the third stage, 300 questionnaires were distributed, a total of 268 questionnaires were collected for self-evaluation of CWB. The questionnaire was matched according to the last four digits of the mother’s telephone number filled in by the respondents, eliminating questionnaires with mismatched codes. Finally, 251 valid questionnaires were obtained (84% response rate). The respondents were given a chance to win a lottery, and participants in each investigation stage were rewarded with no less than 2 dollars. Among the respondents, 68% were women, and their ages ranged from 26 to 55 (94%). Most employees were married (83%) and had a bachelor’s degree (72%). Overall, 95% of employees worked in the hospital for 1?10 years.

### Measures

This study used a relatively mature scale in related fields. The scales were administered to the participants in Chinese. We followed back-translation to translate the scales from English to Chinese and back. The questionnaire used a 5-point Likert scale, where 1 indicates “strongly disagree” and 5 means “strongly agree.”

### Family Supportive Leadership

Family supportive leadership was measured using the 4-item scale developed by Hammer et al. (2009). Sample items are “My leader demonstrates effective behaviors in how to juggle work and non-work balance” and “My leader thinks about how the work in my department can be organized to jointly benefit employees and the company” (α = 0.74).

### Personal Life Attribution

Personal life attribution was measured using the 4-item scale developed by Leslie et al. (2012). Sample items are “My leader provides family support behavior because I have obligations in my personal life that need to be fulfilled” and “My leader provides family support behavior to better meet my responsibilities outside of work” (α = 0.77).

### Work-Family Conflict

Work-family conflict was measured using the 5-item scale developed by Netemeyer et al. (1996). A sample item is “Things I want to do at home do not get done because of the demands my job puts on me” (α = 0.87).

### Moral Disengagement

We used Moore et al. (2012) as a reference to study the scale of moral disengagement, including eight items. Sample items are “It is okay to spread rumors to defend those you care about” and “People should not be held accountable for doing questionable things when they were just doing what an authority figure told them to do” (α = 0.79).

### Counterproductive Work Behavior

Counterproductive work behavior had 19-item adapted from Bennett and Robinson (2000). The sample item included “Said something hurtful to someone at work” (α = 0.89).

### Control Variables

According to relevant literature review and analysis, variables that may affect the research results, such as gender, age, marital status, education level, and weekly work hours, were considered as control variables in this study to better examine the causal relationship between major variables (Asghar et al., 2018; Rofcanin et al., 2020).

### Analytic Strategy

Since employee self-evaluation is a variable in this study, we analyzed the hypothetical model at the individual level. To minimize the impact of common method deviation, we adopted the suggestions provided by Podsakoff et al. (2003), including extending the interval of investigation, placing relevant variables in different investigation stages, and randomizing the order of scale items. The confirmatory factor analysis results of this study show that our construction has good convergence validity, indicating that the common method deviation of this study is not a problem.

SPSS26.0 was used for multiple linear regression analysis to preliminarily test the direct and moderating effects among variables. Specifically, we used SPSS26.0 to test the effect of moral disengagement on CWB (Hypothesis 1), that of work-family conflict on moral disengagement (Hypothesis 2), that of family supportive leadership on work-family conflict (Hypothesis 3), and the moderating role of personal life attribution (Hypothesis 5). To verify the serial mediation model proposed in this study (Hypothesis 4), we used Model 6 of process macro for multiple linear regression analysis. Additionally, we used PROCESS Model 85 to test for moderated mediation effects (Hypotheses 5). We calculated the 95% confidence interval (CI) using the parameter bootstrapping (5000 repetitions) in the process macro of Hayes (2013). If the CI of the effect excluding zero, the proposed indirect effect hypothesis will be supported. To test the moderating effect of the model, we computed the index of moderated mediation for the specific indirect effects (refer to Hayes, 2013 for specific methods).

## Results

### Confirmatory Factor Analysis

We used Mplus8.0 for confirmatory factor analysis to measure the convergence validity of five variables at the individual level (see Table 1). Since CWB has lengthy factor scale structure, to prevent non-convergence issues, to reduce the number of observed indicators, and improve their indicators, we used item parceling method (Takeuchi et al., 2015; Keem et al., 2018). We paired items with highest and lowest loadings to obtain the average value to form a new indicator. Finally, the 19-item of CWB scales were packaged into a 10-item scale. Table 1 shows that the five-factor model had the best fit (χ^2^ = 505.57, df = 314, χ^2^/df = 1.61; RMSEA = 0.06; CFI = 0.93; TLI = 0.92, SRMR = 0.06), which was significantly better than other models, for instance, the four-factor Model A(χ^2^ = 670.84, df = 318, χ^2/^df = 2.11, RMSEA = 0.07, CFI = 0.87, TLI = 0.85, SRMR = 0.07), the three-factor Model B(χ^2^ = 1247.86, df = 321, χ^2/^df = 3.89, RMSEA = 0.11, CFI = 0.65, TLI = 0.62, SRMR = 0.11) and the single-factor model (χ^2^ = 1708.05, df = 324, χ^2^/df = 5.27, RMSEA = 0.14, CFI = 0.48, TLI = 0.44, SRMR = 0.14). Statistical analysis shows that the five variables in this study represent different constructs.

**TABLE 1:** Results of confirmatory factor analysis (*N* = 251).

Model	χ^2^	*df*	χ^2^/*df*	RMSEA	CFI	TLI	SRMR
Five-factor model	505.57	314	1.61	0.06	0.93	0.92	0.06
Four-factor model	670.84	318	2.11	0.07	0.87	0.85	0.07
Three-factor model	1247.86	321	3.89	0.11	0.65	0.62	0.11
Two-factor model	1540.51	323	4.77	0.13	0.54	0.50	0.13
Single-factor model	1708.05	324	5.27	0.14	0.48	0.44	0.14

### Test of Common Method Bias

In this study, Harman single-factor tests were used to determine common method bias. The amount of variation explained by the first principal component when not rotated was 20.52% (less than 40%), and no single factor explained most of the variance, indicating that the potential impact of common methodological biases in this study is not serious.

### Descriptive Statistics Analysis

Table 2 lists the mean, standard deviations, and correlation coefficients of the study variables. Family supportive leadership was negatively correlated with work-family conflict (*r* = –0.18, *p* < 0.01), work-family conflict was positively correlated with moral disengagement (*r* = 0.22, *p* < 0.01), and moral disengagement was significantly positively correlated with CWB (*r* = 0.46, *p* < 0.01). In general, the descriptive statistical analysis provides a necessary basis and premise for the following hypothesis testing.

**TABLE 2:** Descriptive statistics and correlation analysis (*N* = 251).

Variables	Mean	SD	1	2	3	4	5	6	7	8	9
1. Gender											
2. Age	2.23	0.90	0.03								
3. Marital status	2.20	0.96	0.06	0.50**							
4. Education level	2.84	0.61	0.04	–0.04	0.01						
5. Weekly work hours	2.61	0.69	–0.09	0.06	0.14*	–0.09					
6. FLS	3.68	0.71	0.01	0.15*	0.24**	–0.01	0.11				
7. Personal life attribution	3.74	0.75	–0.07	0.09	0.07	0.02	0.12	0.56**			
8. Work-family conflict	2.91	0.98	–0.05	–0.01	0.08	–0.01	0.14*	−0.18**	−0.24**		
9. Moral disengagement	2.01	0.63	−0.14*	–0.02	–0.09	0.01	–0.1	−0.18**	−0.18**	0.22**	
10. CWB	1.96	0.56	−0.14*	–0.01	–0.04	–0.06	–0.08	−0.25**	−0.23**	0.14*	0.46**

N = 251; * p < 0.05; ** p < 0.01. FLS = family supportive leadership, CWB = counterproductive work behavior.

### Hypothesis Testing

When gender, age, marriage, education level, and working hours per week of employees were controlled (see Table 3), moral disengagement positively correlated with CWB (M6: *B* = 0.396, SE = 0.051, *P* < 0.001), supporting Hypothesis 1. Moreover, work-family conflict positively correlated with employee moral disengagement (M3: *B* = 0.156, SE = 0.04, *P* < 0.001), supporting Hypothesis 2. Family supportive leadership was negatively correlated with work-family conflict (M1: *B* = –0.301, SE = 0.087, *P* < 0.01); thus, Hypothesis 3 was validated and supported.

**TABLE 3:** Results of hierarchical regression analysis.

Variables	Work-family conflict		Moral disengagement			CWB					
	M1	M2	M3	M3	M4	M5	M6	M7	M8	M9	M10
Gender	–0.072	–0.112	–0.182*	–0.171*	–0.202*	–0.153*	–0.082*	–0.149*	–0.086	–0.086	–0.173*
Age	–0.059	–0.042	0.023	0.029	0.032	0.012	–0.002	0.015	0.003	0.004	0.021
Marital status	0.147*	0.151*	–0.029	–0.067	–0.027	0.017	0.007	–0.008	0.027	0.026	0.023
Education level	–0.005	–0.01	0.001	0.001	–0.004	–0.055	–0.056	–0.055	–0.055	–0.055	–0.06
Weekly work hours	0.207*	0.211*	–0.082	–0.123*	–0.080	–0.056	–0.035	–0.068	–0.026	–0.027	–0.051
FLS	–0.301**	–0.239*	–0.145*		–0.115	–0.197***		–0.179***	–0.143**	–0.141**	–0.19**
Personal life attribution		–0.273**			–0.138*						–0.127*
Work-family conflict				0.156***				0.058		0.007	
Moral disengagement							0.396***		0.37***	0.37***	
FSL × Personal life attribution		–0.278**			–0.147*						–0.188**
F	3.28**	4.48***	2.73*	4.26***	3.30**	4.01**	11.59***	3.84**	11.74***	10.23***	5.23***
R^2^	0.075	0.129	0.063	0.093	0.098	0.09	0.222	0.1	0.25	0.25	0.15
△R^2^	0.046	0.1	0.026	0.056	0.061	0.06	0.192	0.07	0.22	0.22	0.12

N = 251; M = Model; * p < 0.05, ** p < 0.01, *** p < 0.001. FLS = family supportive leadership. CWB = counterproductive work behavior. Variables involved in the product term were mean-centered.

Hypothesis 4 predicts that family supportive leadership has a serial mediating effect on CWB through work-family conflict and moral disengagement. As shown in Table 4, the total indirect effects of family supportive leadership on CWB is –0.055, and the CI under 95% is [–0.106, –0.014], excluding 0, indicating that the total indirect effects of family supportive leadership on counterproductive work behavior is significant. The effect of family supportive leadership on employees’ CWB through work-family conflict was –0.002, and the CI under 95% was [–0.025, 0.021], including 0, indicating that the mediating effect of work-family conflict was not significant (see M1 in Table 4). The mediating effect of family supported leadership on employees’ CWB through moral disengagement was –0.038, and the CI under 95% was [–0.084, –0.003], excluding 0, indicating that moral disengagement had a significant mediating effect (see M2 in Table 4). The specific serial effect of family supportive leadership on employees’ CWB through work-family conflict and moral disengagement was –0.016, and the CI under 95% was [–0.034, –0.004], excluding 0, indicating that the serial mediation effect was significant (see M3 in Table 4), supporting Hypothesis 4.

**TABLE 4:** Results of serial mediation effect test.

Indirect effects of X on Y	Effect	Boot SE	95%CI
TOTAL	–0.055	0.023	[–0.106, –0.014]
M1: family supportive leadership→ work-family conflict →CWB	–0.002	0.011	[–0.025, 0.021]
M2: family supportive leadership→ moral disengagement →CWB	–0.038	0.021	[–0.084, –0.003]
M3: family supportive leadership→ work-family conflict→ moral disengagement→ CWB	–0.016	0.008	[–0.034, –0.004]

*The number of bootstrap samples for the bias-corrected interval is 5,000. CWB = counterproductive work behavior. CI = Confidence Interval.*

Hypothesis 5 predicts that personal life attribution will moderate the relationship between family supportive leadership and work-family conflict, moral disengagement, and CWB, such that the relationship would be stronger for employees with high personal life attribution. As shown in Table 3, the interaction between family supportive leadership and personal life attribution predicted work-family conflict (*B* = –0.278, SE = 0.09, *p* < 0.01; see M2 in Table 3), that between family supportive leadership and personal life attribution predicted moral disengagement (*B* = –0.147, SE = 0.06, *p* < 0.05; see M4 in Table 3), and that between family supportive leadership and personal life attribution predicted CWB (*B* = –0.188, SE = 0.05, *p* < 0.01; see M10 in Table 3).

**FIGURE 2:**
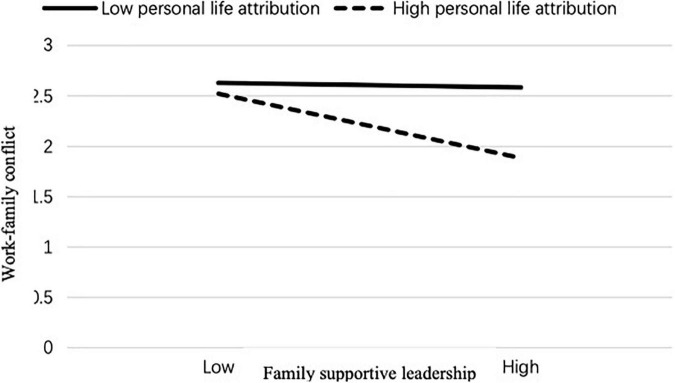
Personal life attribution moderates the relationship between family supportive leadership and work-family conflict.

**FIGURE 3:**
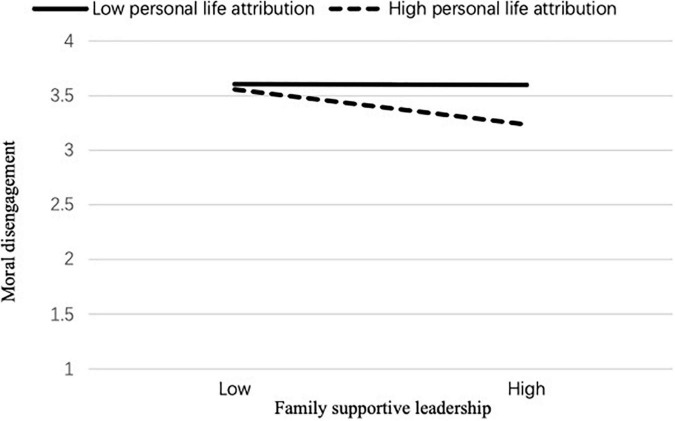
Personal life attribution for family supportive leadership moderates the relationship between family supportive leadership and moral disengagement.

**FIGURE 4:**
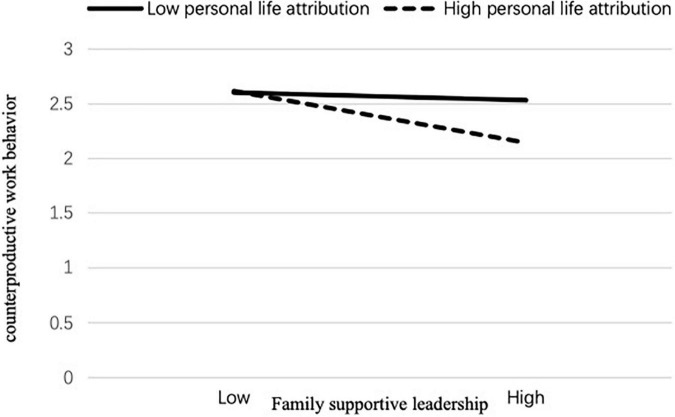
Personal life attribution for family supportive leadership moderates the relationship between supportive leadership and counterproductive work behavior. Five-factor model: family supportive leadership; personal life attribution; work-family conflict; moral disengagement; CWB. Four-factor model: family supportive leadership; personal life attribution; work -family conflict; moral disengagement + CWB. Three-factor model: family supportive leadership; personal life attribution; work -family conflict + moral disengagement + CWB. Two-factor model: family supportive leadership; personal life attribution + work-family conflict + moral disengagement + CWB. Single-factor model: family supportive leadership + personal life attribution + work -family conflict + moral disengagement + CWB.

Simple slope analysis was performed to demonstrate further the moderating effect of personal life attribution on family supportive leadership and work-family conflict. Simple slope analyses showed that the effect of family supportive leadership on work-family conflict was stronger for employees with high personal life attribution (*B* = –0.45, *t* = –4.67, *p* < 0.01) than those with low personal life attribution (*B* = –0.03, *t* = –0.20, *p* = ns; see Figure 2). Moreover, the effect of family supportive leadership on moral disengagement was stronger for employees with high personal life attribution (*B* = –0.22, *t* = –3.59, *p* < 0.05) than those with low personal life attribution (*B* = –0.01, *t* = –0.12, *p* = ns; see Figure 3). Finally, the effect of family supportive leadership on CWB was stronger for employees with high personal life attribution (*B* = –0.28, *t* = –6.34, *p* < 0.001) than those with low personal life attribution (*B* = –0.1, *t* = –1.08, *p* = ns; see Figure 4). Therefore, Hypothesis 5 was supported.

To further verify the moderated mediating effect, this study used Model 85 in the PROCESS program developed by Hayes (2013) to test the indirect effects of the research model (as shown in Figure 1). We computed the index of moderated mediation for the specific indirect effects.

Regarding the index of moderated mediation for M1 in Table 4, personal life attribution moderated the indirect effect of family supportive leadership on CWB through work-family conflict, such that the indirect effect would be more substantial for employees with high personal life attribution. The findings showed that the index of moderated mediation was not significant (index = 0.004, boot SE = 0.011, 95%CI = [–0.017, 0.028]), indicating that the moderated mediating effect is not established. Regarding the index of moderated mediation for M2 in Table 4, personal life attribution moderated the indirect effect of family supportive leadership on CWB through moral disengagement, such that the indirect effect would be stronger for employees with high personal life attribution. Results showed that the index of moderated mediation was significant (index = –0.039, boot SE = 0.022, 95%CI = [–0.089, –0.001]), indicating that the moderated mediating effect was established. Regarding the index of moderated mediation for M3 in Table 4, personal life attribution moderated the indirect effect of family supportive leadership on CWB through work-family conflict and moral disengagement, such that the indirect effect would be more substantial for employees with high personal life attribution. Results showed that the index of moderated mediation was significant (index = –0.011, boot SE = 0.007, 95%CI = [–0.028, –0.001]), indicating that the moderated mediating effect is established.

## Discussion

From the perspective of COR theory, this study examines a serial mediation model between family supportive leadership and CWB. Through the three-stage follow-up investigation of 251 medical workers, we found that family supportive leadership can reduce more disengagement by reducing work-family conflict and ultimately affect the decision-making mechanism of CWB. Our research confirms that work-family conflict and moral disengagement are important influencing factors linking family supportive leadership and CWB. This study also confirmed that personal life attribution moderated the relationship between family supportive leadership and work-family conflict, Such that the higher the personal life attribution of employees, The stronger the negative relationship between family supportive leadership and work-family conflict, and the more ethical decisions they make in the workplace that align with the company’s norms.

### Theoretical Implications

Our study explores the relationship among family supportive leadership, work-family conflict, moral disengagement and CWB from the perspective of work-family relationship, providing a new perspective on the antecedents of CWB and enriching the research content and scope of COR theory. Therefore, it has a certain theoretical significance. First, our research found that providing support and assistance to employees in their work and family relationships can have positive results on employees’ workplace behavior and reduce psychology and behavior that do not conform to organizational norms and ethics (Newman et al., 2019). This finding explains how and when family supportive leadership can mitigate CWB, providing empirical and methodological support for understanding and reducing unethical behavior in the workplace.

Second, to better weaken employees’ negative psychology and behavior, and improve employees’ enthusiasm and initiative in the workplace, we studied whether the influence of family supportive leadership depends on employees’ cognition and view of leadership behavior, that is, employees’ personal life attribution (Keem et al., 2018). We found that personal life attribution moderated the negative relationship between family supportive leadership and work-family conflict. By focusing on the psychological perceptions of employees with different personal life attributions, our research adds new perspectives on reducing negative psychology and behavior in the workplace.

Third, this study introduces work-family conflict and moral disengagement as possible linkage mechanisms. Previous studies have shown that family supportive leadership can positively influence employees’ turnover intention and Deviant behavior (Kelly et al., 2020). Our study demonstrates empirically the presence of a negative correlation between family supportive leadership and CWB. The results show that family supportive leadership has a positive indirect effect on employees’ work behavior through work-family conflict and moral disengagement, which expands our understanding the process of how family supportive leadership affects employees’ work behavior. However, we could not confirm the mediation of work-family conflict role between family supportive leadership and CWB. Supposedly, this phenomenon is that when employees think of work as interference with family, the organization or leader has the responsibility to help deal with these contradictions.

### Practical Implications

The results of our study prove that family supportive leadership plays a crucial role in influencing employees’ work-family relationships and has a favorable impact on the organization, which is primarily why family supportive leadership has attracted widespread attention. Family supportive leadership is a win-win situation for both the organization and employees. However, if the work and family support measures for employees are not properly used, it may have an extremely negative impact on the development of the organization and the harmony of the team. For example, employees may experience psychological injustice from the organization, and family supportive leadership may increase employees’ CWB. The following strategies are recommended to avoid the aforementioned complications.

First, the organization needs to systematically train relevant leaders on how to react to the problem of employee’s work and life, understand resources in and around the organization, and reduce negative workplace behavior by helping employees cope with challenges at work and home. Recent studies have found that training leaders on family support behavior can improve supervisors’ management ability, modify employees’ behavior and work attitude, and improve employees’ job satisfaction and loyalty. Therefore, organizations should also formulate more humane and flexible policies, so that leaders can make decisions independently according to the actual situation when providing family support behaviors, and provide organizational support for employees when dealing with work and family relationships.

Second, we suggest that managers receive training on how to demonstrate family supportive leadership, including when to provide appropriate help to employees’ work-family relationships, analyze employees’ work-family needs, as well as flexibly design work to achieve employees’ work-family balance. These interventions aim to guide supervisors on managing their subordinates’ work and family needs as well as developing criteria to measure whether their family support behavior influences employees’ work behaviors and attitudes as expected.

Finally, we believe that supervisors also need to be communicative in line with employee requirements to meet their work-family needs and maximize the effectiveness of family support. Among these interventions, clarifying supervisors’ and subordinates’ expectations of family supportive leadership behavior appears to be critical to shape the content of relevant training and human resource policies accordingly, and achieve greater consistency between employees and supervisors.

### Limitations and Future Research

This study has several limitations. First, this study adopted three stages of the data collection method. Hence, this study used self-assessments from employees, leading to common method biases. For future research, we encourage using a hybrid method, such as experience-sampling method and experiment study.

Second, future research needs to enrich the action mechanism and boundary conditions of family supportive leadership; this can be done by exploring the effect of family supportive leadership from the perspective of boundary theory (Pan, 2018; Rofcanin et al., 2020). This study only discusses the serial mediating role of work-family conflict and moral disengagement from the perspective of social exchange theory. Future research should examine the impact of different situational factors, personal factors, and their interactions to comprehensively understand the relationship between family supportive leadership and CWB.

Third, recent studies have explored the double-edged sword effect of different leadership types from different perspectives. The negative effects of family supportive leadership, to some extent, can be analyzed from the perspective of moral licensing theory in the future (Yam et al., 2017). Given the important position and role of leaders in work, discussing the impact of leaders’ behaviors on their own work can better help organizations identify the positive and negative effects of leaders and provide guidance for the intervention of leaders’ behaviors in the workplace (Walsh et al., 2018).

## Conclusion

Counterproductive work behavior (CWB) is one of the common problems in the workplace, which seriously affects the sustainable development of the enterprise and the cohesion of the team. Organizations and managers are also actively looking for ways to solve this problem, so that the enterprise can develop healthily and quickly. Based on the COR theory, this study explored the relationship between family supportive leadership and CWB from the perspective of work-family relationship. Many research results show that work and family pressures have a negative impact on employees’ work and family life. However, they ignore how work and family pressure factors affect work outcomes and performance. By applying a new perspective to study the antecedents of CWB, the empirical results of this study demonstrate the positive role of family supportive leadership in reducing CWB. We hope that our research can help organizations and managers improve management efficiency and means, reduce employees’ pressure from work and family to the greatest extent, give full play to their enthusiasm and creativity, and make more contributions to the development of organizations.

## Data Availability Statement

The raw data supporting the conclusions of this article will be made available by the authors, without undue reservation.

## Ethics Statement

The studies involving human participants were reviewed and approved by Academic Committee of Business School Central University of Finance and Economics. The patients/participants provided their written informed consent to participate in this study.

## Author Contributions

All authors listed have made a substantial, direct, and intellectual contribution to the work, and approved it for publication.

## Conflict of Interest

The authors declare that the research was conducted in the absence of any commercial or financial relationships that could be construed as a potential conflict of interest.

## Publisher’s Note

All claims expressed in this article are solely those of the authors and do not necessarily represent those of their affiliated organizations, or those of the publisher, the editors and the reviewers. Any product that may be evaluated in this article, or claim that may be made by its manufacturer, is not guaranteed or endorsed by the publisher.
